# Transverse differences between cleft lip and palate and non-cleft palate with skeletal Class III malocclusion using buccolingual inclination: a cone-beam computed tomography retrospective study

**DOI:** 10.1186/s12903-022-02675-4

**Published:** 2022-12-22

**Authors:** Jiabei He, Lihua Jiang, Shaohua Song, Shuixue Mo

**Affiliations:** 1 Department of Orthodontics, College & Stomatology Hospital, Guangxi Medical University, Nanning, Guangxi, People’s Republic of China; 2 Guangxi Key laboratory of Oral and Maxillofacial Rehabilitation and Reconstruction, Guangxi Medical University, Nanning, Guangxi, People’s Republic of China

**Keywords:** Cleft lip and palate, Buccolingual inclination, Cone-beam computed tomography

## Abstract

**Background:**

The purpose of this study was to evaluate the differences between buccolingual inclination (BI) of maxillary posterior teeth in patients with cleft lip and palate (CLP) and non-cleft palate with skeletal Class III malocclusion. We propose a method of maxillary expansion which is more suitable for patients with CLP.

**Methods:**

For this retrospective study, 40 patients with CLP and 21 patients with skeletal Class III malocclusion were selected. The CLP group was divided into the unilateral cleft lip and palate (UCLP) and bilateral cleft lip and palate (BCLP) groups. The BI of the maxillary first premolar (BI4), maxillary second premolar (BI5) and first molar (BI6) were measured using cone-beam computed tomography, and the differences between them were compared and analyzed by Student’s t-test.

**Results:**

There were significant differences between cleft side BI4 and non-cleft side BI4 in the UCLP group, BI5 in the BCLP group, BI4 and BI5 in all CLP groups and the skeletal Class III malocclusion group. BI6 was similar across all three groups.

**Conclusions:**

The premolars of patients with CLP do not exhibit the same regularity as those with Class III malocclusion; this may be related to surgical scarring of the cleft palate. Greater attention should be paid to the correction of BI in the maxillary expansion of patients with CLP.

## Introduction

Maxillary hypoplasia has become a prevalent deformity that is often caused by cleft palate repair surgery and abnormal maxillary development [1, 2]. Improvement of this developmental deficit requires maxillary expansion therapy, which is one of the most common treatment protocols for expanding a transversally narrow maxilla. Researchers have also applied this treatment approach to patients with cleft lip and palate (CLP) who have a similar clinical presentation as patients with skeletal Class III malocclusion [3, 4] and concluded that other types of arch expanders achieve effective results in patients with CLP, and also present improvements in other areas such as ear function and airway activity [5].

However, different expanders and methods of expansion can achieve different outcomes on the mandible and teeth. Although patients with Class III and CLP share similarities in terms of clinical symptoms, there are differences in maxillary and dental arch width, which are significantly smaller in patients with CLP than in patients with non-CLP Class III [6, 7]. A clearer understanding of the causes of width irregularities in both types of patients is needed to better apply more appropriate arch expanders. It cannot be concluded whether the cause of the reduced width is due to the underdevelopment of the jaw or an abnormality in the angle of the teeth in the jaw simply by using plaster casts and cephalometric measurements.

Cone-beam computed tomography (CBCT) is now the most commonly used auxiliary examination method; it provides a better indication of the relationship between the teeth and the alveolar bone in the posterior region than cephalometric and plaster models. Therefore, based on CBCT, buccolingual inclination (BI) is a better index to evaluate the degree of posterior tooth inclination. It is key to the establishment of ideal occlusal, and is related to the intraoral pressure, position of the tongue, and strength of the tongue and of the buccal muscles [8]. The BI of the posterior teeth of these patients is related to the sagittal relationship, the lateral relationship, and even whether the face is symmetrical [9, 10]. These studies illustrate the “compensation mechanism” of teeth [11]. To a certain extent, teeth can compensate for lateral underdevelopment of the jaw [12]. To date, no studies have measured BI values of posterior teeth in patients with CLP, to investigate whether there are differences in BI values between patients with different types of CLP, or between patients with CLP and patients with non-CLP skeletal Class III malocclusion [9, 13]. Therefore, the purpose of this study was to compare the BI of patients with CLP and those with non-CLP skeletal Class III malocclusion using CBCT. Due to the presence of fissures and surgical scarring in CLP patients, our null hypothesis is that posterior tooth compensation is not significantly different in CLP patients and non-CLP skeletal class III patients, as reflected by the lack of difference in posterior tooth BI values between the two groups.

## Materials and methods

### Ethics committee

The retrospective nature of the study predetermined the sample size. Thus, this study included 40 CLP patients and 21 skeleton Class III malocclusion who were treated at the Department of Orthodontics, Stomatology Hospital, from 2016 to 2021. The study was approved by the university’s ethics review committee (S2021087).

### Inclusion and exclusion criteria

The inclusion criteria were as follows: (1) patients diagnosed with non-syndromic unilateral or bilateral CLP (UCLP or BCLP, respectively); (2) patients with skeletal Class III malocclusion (ANB < 0° or Wits appraisal ≤ − 3.6 mm); (3) maxillary premolars and first molars erupted in occlusion; (4) no previous orthodontic treatment. Exclusion criteria included: (1) CLP with associated syndromes; (2) periodontal disease; (3) history of previous maxillary expansions, maxillary protractions, or secondary alveolar bone grafting. The surgical approach for CLP patients remained consistent within each group. The control group patients were subject to the same inclusion and exclusion criteria.

### CBCT process

CBCT images were obtained using an i-CAT Scanner (Imaging Sciences International Inc, Hatfield, Pennsylvania, United States) set as follows: 16 × 13 cm field of view, 120 kV, 7 mA, with axial slice thickness of 0.25 mm. Each patient was subjected to collection of CBCT image data at the first visit. These were analyzed using Dolphin 3D Imaging software (version 11.8; Dolphin Imaging and Management Solutions, Chatsworth, Calif) in DICOM format.

### Determination of reference plane and measurement of the BI value

**Fig. 1:**
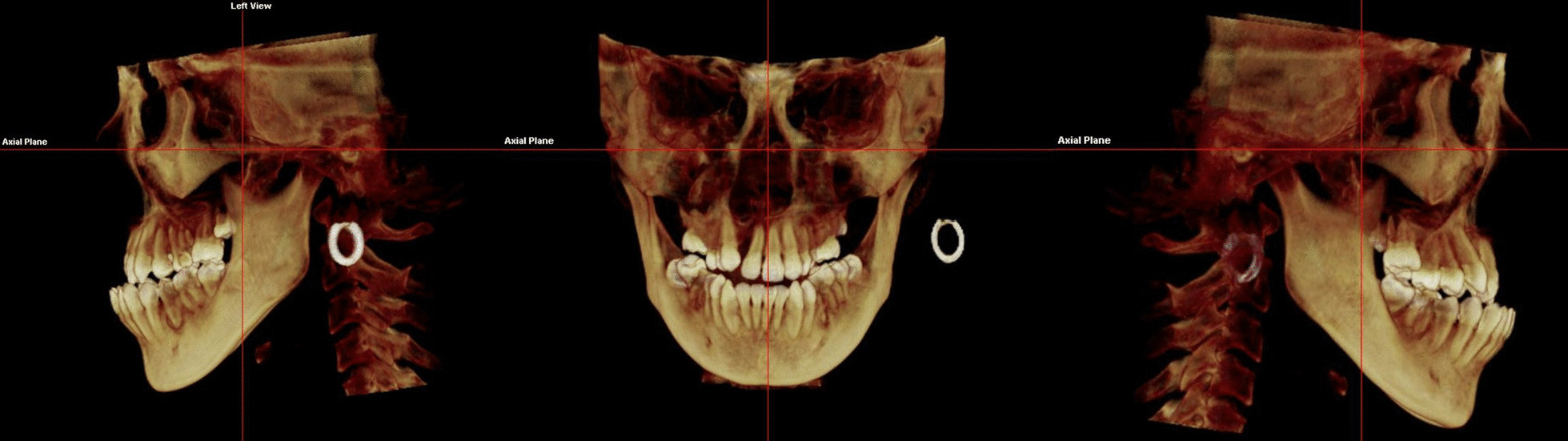
Definition of landmarks and reference planes using Dolphin software. The coincident sagittal and Frankfurt planes were used as the reference planes for head position

**Fig. 2:**
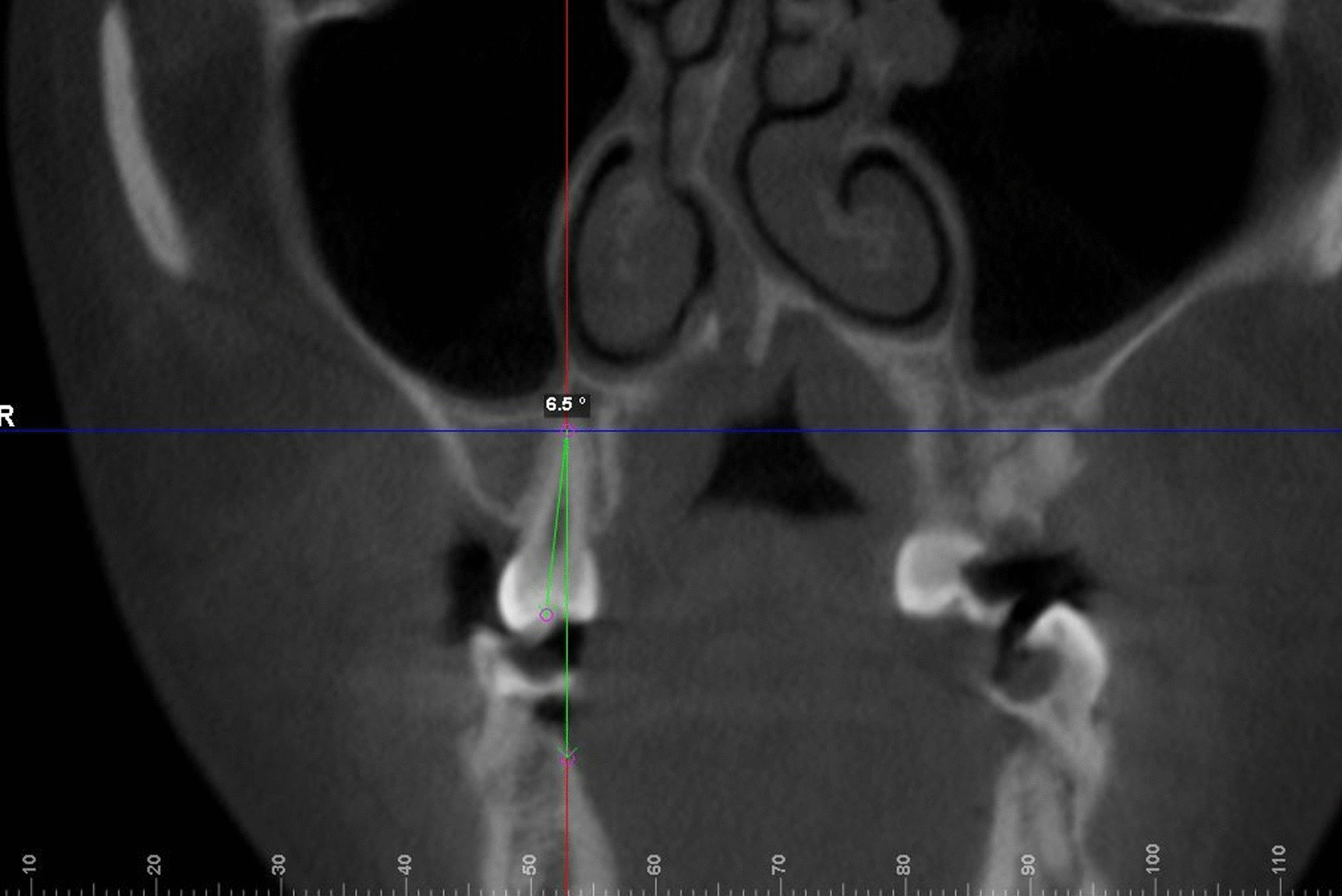
Method for measuring BI value of premolars

**Fig. 3:**
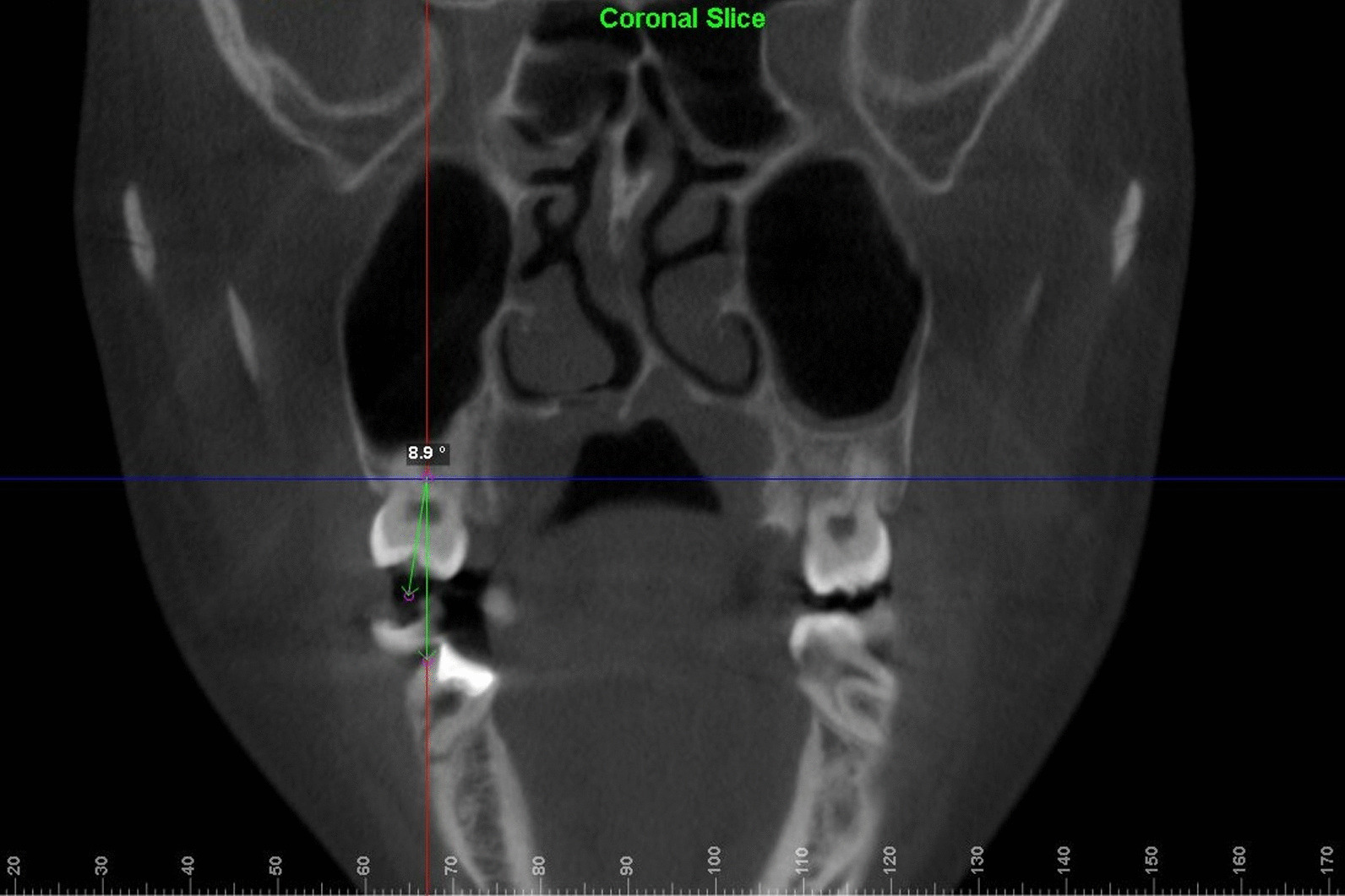
Method for measuring BI value of molars

Dolphin 3D mode was used to locate the landmark. Using click orientation, the left and right view could be selected, and the three-dimensional model could be rotated to identify the patient's Frankfurt plane; the coincident sagittal and Frankfurt planes were used as the reference planes for head position. The corresponding coronal and axial surfaces were used as the measurement planes for BI values. Landmarks and reference planes were defined and are shown in Table 1 and Fig. 1. [9, 14] Teeth inclined to the buccal side have a positive angle, while teeth inclined to the lingual side have a negative angle. Examples of measurements can be seen in Figs. 2 and 3. All BI on the CBCT images were measured and recorded by two researchers. The level of intra-observer agreement for anatomical measurements was assessed using the intraclass correlation coefficient.

**Table 1:** Definition of long-axis planes and lines of maxillary posterior teeth

Variable/tooth	Definition
*Long-axis plane of teeth*	
Maxillary premolar plane	Measuring plane passing through the central fossa and furcation (multirooted) or root apex (single-rooted) of the premolar
Maxillary first molar plane	Measuring plane passing through the central fossa and trifurcation of maxillary first molar
*Long axis of teeth*	
Maxillary premolar	Line passing through the central fossa and furcation (multirooted) or root apex (single-rooted)
Maxillary first molar	Line passing through the central fossa and furcation

### Statistical analysis

The collected data were analyzed using the Kolmogorov–Smirnov test to determine the distribution of each variable. Normally distributed data (*P* < 0.05) are described as mean and standard deviation (X ± s), while non-normally distributed data are reported as median and quartile interval (M ± Q). After measuring all the BI of the maxillary first premolars (BI4), maxillary second premolars (BI5) and first molars (BI6), we examined the differences between the following three groups: the cleft side and the non-cleft side of the UCLP group; the UCLP group and the BCLP group; and each tooth class in the CLP and Class III groups. Student’s t-test was used to evaluate the differences between each group of variables.

## Results

### Patient characteristics

For this retrospective study, 40 patients with CLP and 21 patients with skeletal Class III malocclusion were selected. The CLP group was divided into the UCLP and BCLP groups. The sex and average age of all patients are shown in Table 2.

**Table 2:** Patient characteristics in each group

Groups	Male	Female	Age (X ± s)	*P*-value
UCLP	18	8	12.3 ± 1.2	*P* > 0.05
BCLP	9	5	12.5 ± 1.8	
Class III	11	6	13 ± 1.3	

### Intra-observer correlation

The intra-observer correlation coefficients for each tooth measurement are shown in Table 3. There was high intra-group consistency between the two observers.

**Table 3:** Intra-observer correlation coefficients of each tooth measurement

Tooth	
UCLP first premolar on cleft side	0.990
UCLP first premolar on non-cleft side	0.971
UCLP second premolar on cleft side	0.826
UCLP second premolar on non-cleft side	0.887
UCLP first molar on cleft side	0.890
UCLP first molar on non-cleft side	0.936
BCLP first premolar	0.920
BCLP second premolar	0.873
BCLP first molar	0.867
Class III first premolar	0.959
Class III second premolar	0.976
Class III first molar	0.960

### Comparison of BI values of bilateral posterior teeth in the UCLP group

The comparison within the UCLP group indicated a significant difference between BI4 on the cleft and non-cleft side, but there was no significant difference between BI5 and BI6 on the cleft and non-cleft side. Cleft side premolars were more lingually inclined than non-cleft side premolars. (Table 4).

**Table 4:** Comparison of BI of maxillary posterior teeth between the cleft and non-cleft side in the UCLP group

Tooth	BI (X ± s)	*P*-value
First premolar on cleft side	− 6.10 ± 11.78	0.00
First premolar on non-cleft side	3.50 ± 6.60	
Second premolar on cleft side	− 6.96 ± 9.32	0.13
Second premolar on non-cleft side	− 2.50 ± 9.56	
First molar on cleft side	9.92 ± 4.70	0.6
First molar on non-cleft side	9.01 ± 7.39	

### Comparison of BI values between the UCLP group and BCLP group

Comparison within the BCLP group indicated no statistical differences between bilateral BI4 and BI5, so the bilateral results were combined for the statistical analysis. The value of BI4 was negative and that of BI5 was positive. There was a very marked difference between them.

We found that the second premolar of the UCLP group was more buccally inclined than that in the BCLP group. Differences still existed between the two groups. (Table 5).

**Table 5:** Comparison of BI of maxillary posterior teeth in the BCLP and UCLP groups

Tooth	BI (X ± s)	*P*-value (compared with UCLP)
First premolar	− 6.76 ± 10.13	0.90
Second premolar	2.65 ± 8.11	< 0.01

### Comparison of BI values between CLP group and Class III group

In the comparison of the CLP and Class III groups, except for the second premolars in the BCLP group, the premolars in the Class III group show buccal incline, which was significantly different from the BI of the premolars in the CLP group. Conversely, there was almost no difference in the BI of the molars (Table 6).

**Table 6:** Comparison of BI of maxillary posterior teeth between the cleft group and skeletal Class III malocclusion group

Tooth	BI (X ± s)	Compared with	*P*-value
First premolar	6.09 ± 5.73	UCLP BI4 cleft side	< 0.01
		UCLP BI4 non-cleft side	0.1
		BCLP BI4	< 0.01
Second premolar	5.73 ± 5.29	UCLP BI5 cleft side	< 0.01
		UCLP BI5 non-cleft side	< 0.01
		BCLP BI5	0.1
First molar	10.46 ± 6.33	UCLP BI6 cleft side	0.13
		UCLP BI6 non-cleft side	0.3
		BCLP BI6	0.5

## Discussion

Secondary deformities in infants with CLP are gradually observed in adolescence with growth and development; these deformities seriously affect their masticatory function and appearance. At present, most researchers believe that surgery is the main cause of maxillary deformities in patients with CLP [17]. However, others believe that the cause of maxillary dysplasia in patients with CLP is the cumulative effect of fissures [18]. Very few studies have investigated the compensation of teeth with CLP. A study evaluating alveolar bone and dentition before orthognathic surgery showed that the tooth and bone adaptability of patients with CLP is concentrated on the anterior tooth area. It did not discuss premolar compensation [21]. It is worthwhile to investigate how the maxillary premolar, as the anterior retainer of the maxillary fixed expander, is affected by CLP. There were several innovative aspects in this study. To date there have been no studies on the adaptability of maxillary premolars in patients with CLP, and whether there is a difference in adaptability between UCLP and BCLP is also a question worth exploring. In addition, the results of the study provide some support for surgical scarring causing maxillary deformity. In this study, we explored the mechanism of posterior tooth compensation of patients with CLP from the perspective of BI. The data in the groups revealed statistically significant differences and. Consequently, the null hypothesis was rejected.

In the comparison between the CLP and Class III groups, we noted a significant difference in the inclination of the maxillary premolars, which was mainly negative in patients with CLP. The lateral compensation observed in Class III patients is not well defined in CLP patients. We speculate that this can be attributed to postoperative scarring. After cleft palate surgery, the scar tissue fills the defective bone suture, and the thick collagen fibers connect with the periodontal ligament fibers through the palate, pulling the teeth to a lingual inclination, resulting in the measurement of a negative angle [15]. Moreover, this kind of scar disrupts the balance of internal and external pressure on the teeth, in most cases making it impossible for them to achieve the same occlusion as Class III patients through buccal tilting. The effect of scarring on the lateral development of patients with CLP can be demonstrated by examining the inclination of the posterior teeth.

We observed interesting results in the premolars of the UCLP group. The first premolars on the cleft side were more lingual than the premolars on the non-cleft side. This finding does not contradict the results of our study. Previous plaster and digital model measurements have shown that the arch narrowing of patients with cleft palate is mainly reflected in the premolars, but the measurement of line distance is the result of a pair of teeth and cannot reflect the inclination of a single tooth. Through analysis of the angle of each tooth, we suggest that lingual inclination of the cleft teeth is the main reason for the narrowing of the dental arch [6]. In fact, the severe deformities on the cleft side of patients with CLP not only appears on the dental arch, but differences in nasal patency between the two sides of patients with UCLP have also been reported in nasal airway studies [16]. Nasal volume on the cleft side is smaller than that on the non-cleft side [17]. The hard palate is the anatomical structure between the nasal cavity and the alveolar bone. The appearance of these two deformities further demonstrates the effects of surgical scarring on maxillary deformities.

In the comparison of bilateral second premolars, there were no significant differences, although the *P*-value was small, which may be related to the small sample size of our study. In addition, our study included patients with caries or loss of the second premolars. The angles of these teeth could not be measured. Individuals with CLP may have a higher incidence of dental caries, which could be related to poor cleanliness around the cracks, irregular maxillary dentition, and longer time between eating and brushing [18, 19].

Our study also provided additional interesting findings. For instance, there were distinct differences between BI4 and BI5. The inclination of BI4 was negative while that of BI5 was positive. Because there were no differences in the left and right data of these tooth classes, we merged the data and analyzed the results. Combined with previous first premolar analysis, this may mean that the traction of the scar on the teeth may be obvious in the anterior segment but will be hardly visible on the posterior side. The maxillary first premolar is located at the junction of the anterior scar and the middle palatal suture scar, and the effect is greater than that in the second premolar; the effect weakens progressively in teeth positioned further away from the scar.

The BI values in all groups of first molars were very similar. The tooth compensation in the molar area seemed normal. Plenty of literature has been published, but there is no good explanation for the cause of this phenomenon [6, 20], which may be related to the age of operation and the characteristics of palatal development. Cleft palate repair generally requires a relaxation incision of the whole palate, and the back of the relaxation incision corresponds to the lingual side of the maxillary first and second deciduous molars [21]. Therefore, the area of scar effect should be the area of the dental arch of deciduous molars and the corresponding area of permanent teeth. The eruption location of the first and second molars is related to the continued development of the maxilla, and the development of the maxillary tubercle provides a location for the eruption of the molars. This part of the area is little or almost unaffected by scarring. This could explain why the replaced permanent premolars are limited by scars while the molars erupting from the back of the arch are not.

While studies have attempted to reduce the impact of surgery on upper jaw development by improving the surgical approach and changing the timing of surgery, this does not seem to significantly improve jaw development disorder [2, 22], which underlines the importance of, orthodontic intervention. Maxillary expansion and protraction are the most effective treatment options for patients with CLP after juvenile surgery [23], with outcomes that not only improve the development of the jaw, but also improve the surrounding structure [5]. Our next study will focus on the BI of maxillary posterior teeth in patients with CLP, a more suitable method of arch expansion is worth exploring. For the first premolar area, the BI value was improved by early arch expansion, and the scar tissue attached to the tooth was loosened. Whether this can promote lateral development of the maxilla is worth exploring. The removable expansion palate is the method of arch expansion to which we pay most attention, because its dental efficacy and easy cleaning represent a novel approach to arch expansion and the oral hygiene requirements of patients with CLP [24].

Our study has limitations. The maxillary second molars of some patients were not measured because they had not erupted. The correlation between the length and depth of surgical scarring and maxillary development needs to be verified by further high-quality research.

## Conclusion

Patients with CLP do not show the same tooth inclination as patients with Class III malocclusion. The scarring of cleft palate surgery not only limits the development of the jaw, but also affects the inclination of some posterior teeth. The posterior teeth near the scar are more inclined to the tongue. When performing maxillary expansion treatment for CLP, we should pay closer attention to the BI value of the posterior teeth and adjust the expansion according to the size of the BI value.

## Data Availability

The dataset used and/or analyzed during the present study available from the corresponding author on reasonable request.
